# Conformational Change of Tetratricopeptide Repeats Region Triggers Activation of Phytochrome-Associated Protein Phosphatase 5

**DOI:** 10.3389/fpls.2021.733069

**Published:** 2021-10-14

**Authors:** Silke von Horsten, Lars-Oliver Essen

**Affiliations:** ^1^Department of Biochemistry, Faculty of Chemistry, Philipps-University, Marburg, Germany; ^2^Center for Synthetic Microbiology, Philipps-University, Marburg, Germany

**Keywords:** phytochrome, protein phosphatase, TPR domain, HDX-mass spectrometry, plantal photoreception

## Abstract

Phytochrome activity is not only controlled by light but also by post-translational modifications, e. g. phosphorylation. One of the phosphatases responsible for plant phytochrome dephosphorylation and thereby increased activity is the phytochrome-associated protein phosphatase 5 (PAPP5). We show that PAPP5 recognizes phospho-site mimicking mutants of phytochrome B, when being activated by arachidonic acid (AA). Addition of AA to PAPP5 decreases the α-helical content as tracked by CD-spectroscopy. These changes correspond to conformational changes of the regulatory tetratricopeptide repeats (TPR) region as shown by mapping data from hydrogen deuterium exchange mass spectrometry onto a 3.0 Å crystal structure of PAPP5. Surprisingly, parts of the linker between the TPR and PP2A domains and of the so-called C-terminal inhibitory motif exhibit reduced deuterium uptake upon AA-binding. Molecular dynamics analyses of PAPP5 complexed to a phyB phosphopeptide show that this C-terminal motif remains associated with the TPR region in the substrate bound state, suggesting that this motif merely serves for restricting the orientations of the TPR region relative to the catalytic PP2A domain. Given the high similarity to mammalian PP5 these data from a plant ortholog show that the activation mode of these PPP-type protein phosphatases is highly conserved.

## Introduction

During evolution plants evolved complex mechanisms to ensure their survival as sessile organisms. Given that sunlight is essential for survival and growth, plants employ a variety of photoreceptors and interconnected signaling cascades to optimize their photosynthetic potential and to synchronize their lifecycles with circadian and seasonal rhythms (Rockwell et al., 2006). As a classical plant photoreceptor phytochromes are photochromic toward red/far-red light and consist of a large (∼120 kDa) apoprotein with a covalently bound phytochromobilin (PΦB). This bilin chromophore enables plant phytochromes to absorb red/far-red light by *cis*-*trans* isomerization along the C15-C16 bond for triggering large structural changes within phytochrome dimers and distinguishing between the photon fluence rate, direction, duration and light quality. In addition, plant phytochromes like phytochrome B (phyB) act as temperature sensors (Jung et al., 2016; Legris et al., 2016), given their high activation energy for thermal dark reversion. Accordingly, these phytochromes are integrators of different environmental cues and trigger numerous developmental processes *in planta* such as seed germination, photomorphogenesis or floral induction (Rockwell et al., 2006; Franklin and Quail, 2010).

When plant phytochromes are photoconverted from their inactive, red-light sensitive P_*r*_ state to the active, far-red sensitive P_*fr*_ state, they are transported into the nucleus, where they can interact with various effectors such as phytochrome interaction factors (PIFs), a class of transcription factors, whose further modification and degradation is controlled by phytochromes (Sakamoto and Nagatani, 1996; Kircher et al., 1999; Yamaguchi et al., 1999; Hisada et al., 2000; Park et al., 2004; Chen et al., 2005). However, PIFs are not the only interaction partners as phytochrome-dependent signal transduction pathways are complex and composed of an intricate network of numerous downstream-signaling components (Quail, 2002). Accordingly, phytochrome-dependent signaling is not only restricted to the cell nucleus, but also occurs in the cytoplasm. These cytosolic signaling routes presumably allow a faster mode of signal transduction, since relocalization of phytochromes into the nucleus requires up to 2 h (Parks and Spalding, 1999).

The N-terminal photosensory module (PSM) of plant phytochromes, which mediates light perception, resembles bacterial and fungal orthologs by consisting of a PAS (Per/Arndt/Sim), GAF (cGMP phosphodiesterase/adenylyl cyclase/FhlA) and PHY (Phy-specific GAF) domain (Anders and Essen, 2015). Plant phytochromes have a unique composition for their C-terminal output module, comprising of two additional PAS domains and a histidine kinase-related domain. Furthermore, they possess an N-terminal extension (NTE) before the PSM, which is especially prominent in phyB and phyD with a length of ∼ 90 amino acids. This extension contains a serine- and glycine-rich region (Mathews, 2010) and is predicted to be largely unstructured. However, recent data indicate a light-dependent interaction with the PSM, as parts of the NTE exhibit light-switchable H/D exchange rates (Von Horsten et al., 2016). Interestingly, one of these parts (S84-K88) corresponds to a tandem of phosphorylation sites in phyB from *Arabidopsis thaliana*, S84 and S86 (Medzihradszky et al., 2013; Von Horsten et al., 2016). Phosphorylation of these sites shows an effect on the binding affinity of phytochromes toward some interaction partners and demonstrate the role of the NTE as a modulatory element for light-dependent phytochrome activity (Casal et al., 2002; Kim et al., 2004).

Whereas the kinase that phosphorylates plant phytochromes has not yet been discovered and an autophosphorylation activity appears to be present as well (Shin et al., 2016), four phosphatases have been identified to affect the phytochromes’ phosphorylation status (Bheri et al., 2020). The flower-specific phytochrome-associated protein phosphatase (FyPP) is named after its involvement in phytochrome-regulated flower formation and belongs to the family of type 6 protein phosphatases (Kim et al., 2002). The second, a type 7 phosphatase (PP7), controls phytochrome activity as well (Genoud et al., 2008). Two further phosphatases are the phytochrome-associated protein phosphatase 2c (PAPP2c), a phosphatase identified to interact with the PHY Domain of PhyA and to dephosphorylate the NTE of both *At*PhyA and *At*PhyB (Phee et al., 2008), and the type 5 serine/threonine protein phosphatase (PAPP5). The latter represents a unique type within the PPP family of serine/threonine phosphatases due to its N-terminal tetratricopeptide repeats (TPR) (Chen et al., 1994). The three repeats form a common α-helical bundle that is connected to the type 2A serine/threonine protein phosphatase (PP2A) domain *via* a long helical linker. A C-terminal inhibitory motif blocks together with the TPR region the active site of the PP2A domain, where two catalytic manganese ions are bound (Yang et al., 2005). PAPP5 is predominantly located in the nucleus but also present at lower levels in the cytoplasm, as the C-terminal motif of PAPP5 contains a putative nuclear localization signal (476–491) (Chen et al., 1994; Zeke et al., 2005). Furthermore, a second splice form of PAPP5 encoding mRNA yields a larger, membrane-localized isozyme of PAPP5 due to the presence of two additional transmembrane helices (De La Fuente Van Bentem et al., 2003). Studies by Ryu et al. have shown that the PP2A domain of PAPP5 dephosphorylates the NTE of *As*PhyA and *At*PhyB, whereas the TPR region binds to the C-terminal histidine kinase-related domain of *As*PhyA (Ryu et al., 2005). The interaction between phytochromes and PAPP5 is thereby light-dependent and occurs preferentially, when the phytochrome adopts its activated P_*fr*_ state. Other functions of the soluble isoform of PAPP5 might be plantal signal transduction via plastid-derived tetrapyrroles (Barajas-Lopez Jde et al., 2013) or karrikins and the unknown endogenous KAI2 ligand (Struk et al., 2021).

Apparently, low *in vitro* activity is the reason for the late discovery of PAPP5 (Chen and Cohen, 1997). Variants of the human *PP5* ortholog without the TPR region or the C-terminal inhibitory motif show an high increase in phosphatase activity. Mutagenesis studies have revealed that especially E76 (*At*PAPP5: E61) of the TPR region (Kang et al., 2001) and the last 13 residues of the C-terminus are essential for blocking the active site (Chen and Cohen, 1997; Sinclair et al., 1999; Kang et al., 2001). Like mammalian PP5, PAPP5 is activated by the polyunsaturated fatty acid arachidonic acid (AA) (Ryu et al., 2005). As higher plants do not produce eicosapolyenoic acids, AA has been postulated to act in plants as an exogenous signaling molecule for stress and host defense (Savchenko et al., 2010).

Next to the absorption of light, phosphorylation/dephosphorylation presents a second pathway to fine-tune the activity of plant phytochromes. From the set of phosphatases responsible for dephosphorylating phytochromes, PAPP5 proves to be an interesting interaction partner since it interacts with both the N- and C-terminus of phytochrome A from *Avena sativa* and *Arabidopsis thaliana* (Ryu et al., 2005). In this report, we investigated *At*PAPP5 and solved its crystal structure, which enabled a molecular understanding of the autoinhibition and the role of its C-terminal motif. Studies addressing the structural changes in *At*PAPP5 upon AA-binding allowed us to create a general activation model for phosphatases of the PP5 subfamily.

## Experimental Procedures

### Cloning, Overexpression, and Purification of *At*PAPP5, *At*PhyB(1–651) and *At*PhyB/S84D and *At*PhyB/S86D Mutants

The DNA fragment encoding isoform 2 of *papp5* from *Arabidopsis thaliana* (UniProtKB: Q84XU2-2; 1-484) was codon-optimized and flanked by *Nde*I and *Sal*l restriction sites (Geneart, Invitrogen Life Technologies). Afterward, the fragment was subcloned into the pET-28a vector (Novagen) using the corresponding restriction sites.

The plasmid carrying the coding sequence for the photosensory module of *Arabidopsis thaliana phytochrome B* (UniProtKB: P14713-1; 1-651) was kindly provided by Andreas Zurbriggen (University Düsseldorf) and subcloned into the pCDF Duet-1 vector (Novagen) by polymerase chain reaction using primers, which introduced *Pci*I and *Pst*I restriction sites. S84D and S86D mutants were generated by QuickChange site-directed mutagenesis (Stratagene). All primers are listed in Supplementary Table 1. Sequences of the resulting plasmids were controlled by dideoxy sequencing (GATC).

Expression was performed in the *E. coli* BL21 Gold (DE3) strain (Novagen). Cultures for *At*PAPP5 overproduction were grown in TB medium containing 35 mg/L kanamycin at 37°C to an OD_595_ of 0.6, the temperature was decreased to 18°C and expression was induced with 50 μM IPTG. After 22 h the cells were harvested by centrifugation (8,200 g, 15 min, 4°C), resuspended in Mn-buffer (50 mM Tris, pH 8.0, 100 mM NaCl, 4 mM MnCl_2_, 0.1 mM β-mercaptoethanol), frozen in liquid nitrogen and stored at −80°C. Bacterial cells were lysed by a French Press cell (AMINCO) and the supernatant was separated by centrifugation (40,000 g, 30 min, 4°C), followed by Ni^2+^-affinity chromatography (HisTrap^TM^ HP column, GE Healthcare) and eluted with TIS buffer (50 mM Tris, 250 mM imidazole pH 7.8, 100 mM NaCl, 4 mM MnCl_2_, 1 mM β-mercaptoethanol). A final purification step was done by size exclusion chromatography (Superdex 200 26/60, GE Healthcare) using 50 mM Tris pH 7.8, 1 mM EDTA, 100 mM NaCl, 4 mM MnCl_2_, 1 mM β-mercaptoethanol as buffer. Before usage of *At*PAPP5 for assays the affinity tag was cleaved off by thrombin (overnight at 4°C) and removed by reversed Ni-NTA.

Overproduction and purification of wild-type *At*PhyB(1-651) and the phosphomimic mutants harboring the PCB chromophore was performed as described before (Von Horsten et al., 2016). Prior to usage samples were centrifuged for 10 min at 4°C and 13,000 rpm.

### Phosphatase Activity Assays

The assay was performed as described by Ryu et al. (2005). In 100 μL reaction mixture, 1 μg *At*PAPP5 in kinase/phosphatase buffer (KP: 25 mM Tris-HCl pH 7.5, 5 mM MgCl_2_, 20 mM Mg(CH_3_COO)_2_, 0.2 mM EDTA, 0.2 EGTA) was used in the absence or presence of arachidonic acid/6% ethanol. The concentration of *p*-nitrophenyl phosphate (pNPP) was varied. After an incubation time of 15 min at 30°C the reaction was terminated by the addition of 900 μL 0.25 M NaOH and the absorbance at 410 nm was measured.

### Microscale Thermophoresis Analysis of AtPAPP5-AtPhyB Interaction

Microscale thermophoresis was conducted to determine the *At*PAPP5 dissociation constant toward the *At*PhyB(1-651) mutants S84D and S86D by using a Monolith NT.115 instrument (NanoTemper). Freshly prepared *At*PAPP5 (50 μM in labeling buffer) was labeled at 8°C overnight using the cysteine-reactive Monolith NT.115 Protein Labeling Kit GREEN MALEIMIDE (NanoTemper) to achieve a 1:1 molar ratio of labled protein to dye. A serial titration from 4 nM to 70 μM of S86D and 2 nM to 29 μM of S84D was prepared and labeled. *At*PAPP5 was diluted to each sample until a final concentration of 50 nM. Afterward 100 μM AA was added and the mixture was incubated for 15 min before measurement. The assay was then transferred into *premium coated* capillaries (NanoTemper) and measured at 25°C. The LED power for each thermophoresis measurement was set to 80% and the laser power to 20%, the heating time was set to 30 s, followed by 5 s of cooling. Binding curves were obtained from the thermophoresis hot to cold ratio. Negative controls were performed without the addition of AA and with *At*PhyB WT under the same conditions mentioned above. Each experiment was repeated in triplicate, analyzed with the *NT Analysis software* (NanoTemper) and the K_*D*_ value was calculated with Origin 9.0 (OriginLab) using a 1:1 binding model.

### Size Exclusion Chromatography

A 25 μM of *At*PAPP5 in KP buffer (25 mM Tris-HCl pH 7.5, 5 mM MgCl_2_, 20 mM Mg(CH_3_COO)_2_, 0.2 mM EDTA, 0.2 EGTA) buffer was injected onto a Superdex 200 10/300 column using an Äkta purifier (GE Healthcare). The setup was repeated after adding 30 μM arachidonic acid. The runs were monitored at 280 nm. The apparent molecular masses of the eluted species were calculated by using a molecular weight standard curve based on a column calibration (LMW kit, GE Healthcare) performed previously.

### Circular Dichroism Spectroscopy of *At*PAPP5

A solution of 40 μM *At*PAPP5 in HEPES buffer (10 mM HEPES pH 8.0, 100 mM NaCl, 4 mM MgCl_2_) with 0 μ, 25, 50, 75, 100, and 150 μM AA, using a 3.2 mM AA stock solution in EtOH were incubated at room temperature for 15 min and measured with a spectropolarimeter J-810 (Jasco) using a 1 mm cuvette. After a baseline correction with the corresponding buffer, which contained an equal concentration of ethanol, the data was averaged over three scans.

### Crystallization and Structure Determination of *At*PAPP5

*At*PAPP5 was transferred into crystallization buffer (10 mM Tris pH 7.5, 100 mM NaCl, 5 mM MnCl_2_), concentrated to 15 mg/mL and crystallization attempts were pipetted with a Honeybee robot system (Zinsser Analytic) using commercially available screens (Qiagen) in a 96-well format. Crystals were grown at 18°C using the sitting-drop method and vapor-diffusion. After 2 weeks, small crystals were found growing in condition C10 from crystal screen Core I (Qiagen). To obtain bigger crystals optimizations were done using streak seeding with crushed crystals from the original hit. The crystallization drop for the optimizations consists of 1 μL protein solution and 1 μL of reservoir solution. For optimization, the hanging-drop vapor diffusion method was used. After 4 weeks, crystals appeared in a condition containing 0.1 M HEPES pH 7.0 and 50% (v/v) PEG4000. These were picked and incubated in cryoprotection buffer (reservoir buffer supplemented with 30% glycerol) prior to freezing in liquid nitrogen.

An *At*PAPP5 dataset was collected at beamline 14.2 of BESSY-II (Berliner Elektronenspeicherring-Gesellschaft für Synchrotronstrahlung, Berlin, Germany) and processed with the XDS (Kabsch, 2010) and CCP4 (Collaborative Computational Project, 1994) package. The structure for the dataset was solved by molecular replacement using PHENIX autosolve (Adams et al., 2010) and 1WAO as search model (Yang et al., 2005). Manual model building was conducted with COOT (Emsley and Cowtan, 2004) and refinement by PHENIX refine (Adams et al., 2010). Figures were created using PYMOL 1.6 (DeLano Scientific).

### Hydrogen Deuterium Exchange-Mass Spectrometry of AtPAPP5

HDX-mass spectrometric analysis *At*PAPP5 (60 μM) with and without arachidonic acid (75 μM) was carried out using a commercial HDX-automation setup (SYNAPT G2-Si, Waters) including a two-arm robotic autosampler (LEAP Technologies), an ACQUITY UPLC M-Class system (Waters) and HDX manager (Waters). *At*PAPP5 samples were transferred by a PD-10 column into a low salt buffer (10 mM Tris pH 8.0, 100 mM NaCl, 4 mM MgCl_2_, 1 mM β-mercaptoethanol) and centrifuged for 10 min at 16,100 g and 4°C. Exchange reactions and analysis were performed as described before (Von Horsten et al., 2016).

**FIGURE 1:**
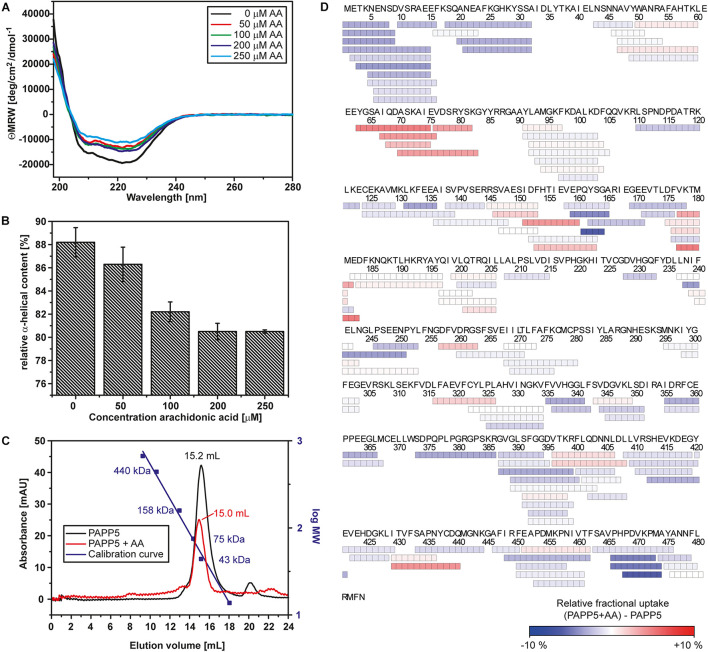
Arachidonic acid activates *At*PAPP5 by altering its structure. **(A)** Far-UV CD-spectrum of *At*PAPP5 in the absence or presence of various concentrations of arachidonic acid (AA). **(B)** Calculated relative alpha-helical contents from the CD measurements presented in a bar chart. **(C)** Size exclusion chromatogramm of *At*PAPP5 in the presence or absence of AA. The addition of arachidonic acid alters the elution volume from 15.18 to 14.96 mL. The molecular masses of calibration standards are plotted using the second logarithmic ordinate. The absorbance was recorded at 280 nm, respectively. **(D)** Coverage map of the HDX-MS analysis for the difference between *At*PAPP5 and *At*PAPP5 with AA after 15 s deuterium incubation. Each box reflects one peptide and the coloring is according to the relative fractional deuterium uptake colored in blue-white-red presentation.

Final assignment of deuterium incorporation was done with DynamX 3.0 (Waters). The minimum peak intensity was set to 10^3^ counts and a peptide length between four and 15 was chosen. Moreover, tolerances of 0.5 min for the retention time and 25 ppm for m/z values were applied for the peptide assignment, generating an overall sequence coverage of 80.0%. 125 peptides were analyzed with an overall redundancy of 3.0 per amino acid. A standard deviation of 4 σ was used to quantify the amount of variation between the repetitions. A coverage map of all peptides is presented in Figure 1D. The results of the analysis were mapped onto the structure of *At*PAPP5.

### Molecular Dynamics Analysis of *At*PAPP5•phyB Peptide Complexes

Molecular dynamics analyses of *At*PAPP5 were done with the GPU-accelerated Amber18 suite (Lee et al., 2018) using the ff19sb force field for the protein (Tian et al., 2020) and the SPC/E water model. pKa values of ionizable residues of *At*PAPP5 at pH 6.5 were estimated by H++. Using B3LYP/6-31G^∗^ model chemistry we calculated parameters for the μ-hydroxyl bridged di-Mn^2+^ center either in its free state or complexed to methyl-phosphate or phosphoserine-comprising phyB peptides by gaussian16 (Frisch et al., 2016) and MCPB.py (Li and Merz, 2016). As start geometry for these QM-calculations we used the di-Mn^2+^ center of human PP5 complexed to the Cdc37 substrate peptide (Oberoi et al., 2016). For MD simulations free *At*PAPP5 was neutralized by 57 chloride and 58 sodium ions (∼0.15 M) and placed in a box with 31,718 water molecules, which equilibrated to box dimensions of 100.6 × 100.8 × 100.5 Å^3^ (Supplementary Table 2). *At*PAPP5 ensembles were minimized, pre-equilibrated as NVT ensembles followed up for 5.5 ns as NpT ensembles at 300 K using 2 fs steps, 10 Å cutoff and a Monte-Carlo barostat. The systems were further equilibrated for 100 ns before generating projection trajectories with lengths of 180–1,000 ns. *At*PAPP5 trajectories were processed and evaluated in jupyter notebooks using pytraj 2.0.5 (Roe and Cheatham, 2013),^1^ AmberTools20 and NGLview 2.7.7 (Nguyen et al., 2018). The total length of four independently generated production trajectories for *At*PAPP5 in the unbound, closed and the phyB S80-T89 phosphorylated peptide states amount to 4 μs for each, for the complex between *At*PAPP5 and the phyB D82-S86 peptide phosphorylated at S84 to 2.88 μs and the complex with phosphoserine to 3.50 μs (Supplementary Table 2). *At*PAPP5-bound peptides were capped at their N- and C-termini by acetyl and methylamine groups, respectively.

## Results

### Activation of *At*PAPP5 by Fatty Acids and Ethanol

For structural analyses, we used recombinant *At*PAPP5 that was produced as histidine-tagged monomer by heterologous expression in *E. coli*. In contrast to previous reports (Ryu et al., 2005) we removed affinity tags for activity measurements.

**FIGURE 2:**
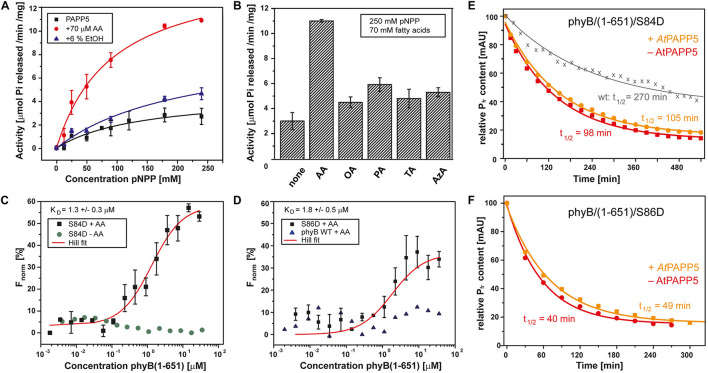
*At*PAPP5 activity and interaction with the phosphomimic mutants S84D and S86D of phytochrome B. **(A)** Phosphatase activity (mean ± SD; n = 3) of *At*PAPP5 in the presence or absence of 70 μM arachidonic acid (AA) or 6% ethanol (EtOH) and various concentrations of the artificial substrate, p-nitrophenyl phosphate (pNPP). **(B)** Influence of fatty acids (70 μM) on the phosphatase activity (mean ± SD; *n* = 3) of *At*PAPP5 in the presence of 250 mM pNPP. The line marks the activity of ethanol alone. OA, oleic acid; PA, palmitic acid; TA, traumatic acid; AzA, azaleic acid. **(C,D)** Microscale thermophoresis experiments show binding (mean ± SD; *n* = 3) of *At*PAPP5 toward *At*PhyB/S86D and *At*PhyB/S84D. Notably, the S84D achieved no saturation. Therefore, this value should only be regarded as approximation. As negative controls the measurement were done either without AA **(C)** or with wild-type *At*PhyB(1-651) **(D)**. The fitting of experimental data assumed a 1:1 binding model. **(E,F)** The dark conversion of the S84D and S86D variants (red), in the presence of AtPAPP5 and AA (orange) and the half-lives (t_1__/__2_) calculated therefrom.

Earlier reports showed that AA activates *At*PAPP5 as well as the human PP5 ortholog (Chen and Cohen, 1997; Skinner et al., 1997; Ryu et al., 2005). To confirm the functionality of recombinant *At*PAPP5, we measured the phosphatase activity by an assay based on para-nitrophenyl phosphate (pNPP) as chromogen. The amount of released phosphate (P_*i*_) corresponds to the formation of para-nitrophenolate that is monitored at 405 nm. The assay was initially conducted for *At*PAPP5 in the presence of 70 μM AA that was dissolved in ethanol. Control reactions were carried out with an equivalent volume of ethanol. Surprisingly, ethanol alone already activates *At*PAPP5 by releasing the blocked active site (Figure 2A). In the presence of AA *At*PAPP5 shows a Michaelis-Menten kinetics for varying pNPP concentrations with a K_*M*_-value of 92.2 mM and a V_*max*_ value of 15 ± 1.1 μmol/mg/mL. These values resemble kinetic constants obtained by Ryu et al. (2005) for a GST-*At*PAPP5 fusion with 100 μM AA (K_*M*_ 160 mM; V_*max*_ 22 μmol/mg/mL). Interestingly, addition of arachidonic acid caused only a doubled turnover rate relative to the ethanol-activated state (V_*max*_ = 8.9 ± 1.9 μmol/min/mg) and a fourfold increase compared to wildtype without any AA and EtOH (V_*max*_ = 3.6 ± 0.7 μmol/min/mg).

We also checked PAPP5 activities in the presence of different fatty acids using saturating substrate concentrations, i.e., 250 mM pNPP (Figure 2B). None of the other fatty acids activated PAPP5 like AA. To further investigate the influence of ethanol on the phosphatase activity, ethanol concentrations were varied between 0 and 90% (v/v). Interestingly, these data show an up to 12-fold boost of PAPP5 activity with 75% ethanol (Supplementary Figure 1), which may reflect either partial unfolding of the TPR region or a disruption of TPR-PP2A domain interface.

### *At*PAPP5 Interacts With the N-terminal Extension of phyB

Structural studies on the catalytic domain of human PP5 complexed to a Cdc37-derived peptide showed that phosphomimetic S→D mutations serve as useful tools for analyzing phosphatase-phosphopeptide interactions (Oberoi et al., 2016). Accordingly, we proved direct interaction between *At*PAPP5 and the NTE of *At*PhyB by generating two phosphomimetic variants of *At*PhyB(1-651), S84D, and S86D. Phosphorylation/dephosphorylation of S84 and S86 in the NTE phyB was shown to be strongly involved in the negative *in vivo* regulation of phyB (Medzihradszky et al., 2013; Nito et al., 2013). We performed sensitive interaction assays by microscale thermophoresis (MST) using *At*PAPP5 that was labeled before with a green-fluorescent dye. After preincubation with AA for 15 min *At*PAPP5 binding was measured against a concentration series with either *At*PhyB/S84D or *At*PhyB/S86D. Since our MST setup uses a green laser for fluorescence detection (λ_*ex*__*c*_: 515–525 nm, λ_*em*_: 650–685 nm), the phytochrome variants were kept in a photodynamical equilibrium with a prevalence of their activated P_*fr*_ state. *At*PAPP5 showed a high affinity interaction to AtPhyB phosphomimic variants with an apparent K_*D*_ of 1.3 ± 0.3 μM for S84D and 1.8 ± 0.5 μM for S86D, respectively (Figures 2C,D). To rule out non-specific binding, the measurements were repeated using recombinant wild-type *At*PhyB(1–651) and resulted in signals not exceeding signal-to-noise ratios. Accordingly, non-specific binding to the photosensory module of *At*PhyB in its non-phosphorylated P_*fr*_ state can be excluded. Furthermore, measurements in the absence of AA resulted in baselines showing the necessity of *At*PAPP5 activation by AA (Figure 2C).

We analyzed the photochemical behavior of our phosphomimic mimics by UV/Vis spectroscopy. Compared to wild-type *At*PhyB (Von Horsten et al., 2016) absorption maxima are bathochromically shifted, especially in the P_*fr*_ form with shifts of 8 nm for the S84D and 5 nm for the S86D variant (Supplementary Figure 2). Furthermore, both phytochrome B variants exhibit an accelerated dark conversion rate (t_1__/__2_ of S84D: 98 min; S86D: 40 min) compared to the photosensory module of wildtype phyB (t_1__/__2_: 270 min; Figures 2E,F; Von Horsten et al., 2016). Addition of *At*PAPP5 and AA induced no further shift of the maxima according to UV/Vis spectra. However, there is a slightly reduced dark conversion rate with half-lives of 105 min for S84D and 49 min for S86D.

### Closed State Structure of *At*PAPP5

We crystallized *At*PAPP5 in a monoclinic crystal form that resulted in a dataset at 3.0 Å resolution. Unfortunately, we could not treat these AtPAPP5 crystals with AA because they rapidly dissolved upon incubation. Molecular replacement using protein phosphatase 5 from *Homo sapiens* (*Hs*PP5; PDB Code 1WAO) as search model (sequence identity: 56% for 484 amino acids) allowed structure determination with two molecules per asymmetric unit. Table 1 shows data collection and refinement statistics of our *At*PAPP5 structure.

**TABLE 1:** Statistics for data collection and refinement of *At*PAPP5.

	*At*PAPP5 (7OBE)
**Data collection**	
Wavelength [Å]	0.91841
Space group	*P* 1 21 1
Cell dimensions	*a* = 51.6 Å, *b* = 103.0 Å, *c* = 95.1 Å; β = 95.6°
Resolution [Å]	34.8–3.0 (3.1–3.0)
Measured and unique reflections	37,619, 19,742
*R*_*merge*_	0.072 (0.240)
*I*/σ(*I*)	13.7 (3.93)
Mosaicity [°]	0.329
Completeness [%]	99.1 (99.6)
Wilson B-factor [Å^2^]	32.3
Solvent content [%]	49.3
Multiplicity	1.9 (1.9)
**Refinement**	
Resolution [Å]	3.0
*R*_*work*_, *R*_*free*_ [%]	0.210; 0.243
Residues	956
Defined in chain A	476 (D9-N484)
Defined in chain B	480 (N5-N484)
Water molecules	21
Heteroatoms	4 Mn^2+^; 2 Cl^–^
r.m.s.d. bonds [Å]	0.582
r.m.s.d. angles [°]	0.003

*Values in parentheses refer to the highest resolution shell.*

**FIGURE 3:**
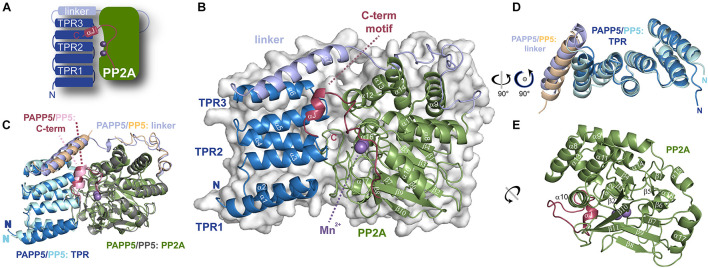
Crystal structure of *At*PAPP5 phosphatase. **(A)** Schematic organization of AtPAPP5. **(B)** The TPR region (blue) is arranged in an α-helical bundle and connected *via* a long linker region (light blue) to the PP2A domain (green), in whose active site two Mn^2+^ ions (purple) are bound. The C-terminal inhibitory motif (raspberry) and the TPR region cover the active site. **(C)** Superposition of *At*PAPP5 and *Hs*PP5 structures (PDB code: 1WAO). **(D)** After superposition on the central TPR2 repeat, the superposed TPR regions show some deviations for the TPR3 repeat and the linker helix. **(E)** The catalytic PP2A domain consists of 11 β-strands arranged as a central β-sandwich that is surrounded by α-helices.

The *At*PAPP5 structure shows the characteristic bilobal shape of a type 5 serine/threonine phosphatase with two manganese ions bound in the active site. Three TPR repeats (TPR1: V10-L42, TPR2: W50-V76 and TPR3: S81-L110) are linked *via* a long helical linker (P115-T173) to the PP2A domain (L174-N484). The TPR repeats of the N-terminal domain consist each of two α-helices as part of a helix-turn-helix motif; these motifs stack to each other causing formation of a superhelical tertiary structure. The last 14 amino acids of the PP2A domain form a separate motif, the C-terminal inhibitory motif that consists of a linker (K471-A476) and a short α-helical segment (αJ, N477-M482; Figure 3A). The strictly conserved PP2A domain adopts a compact α/β fold consisting of eleven α-helices and eleven β-strands, which form together the central β-sandwich (Figure 3E). This sandwich is flanked on one side by three short helices (α9-α11) and on the other side by six helices (α12-α17). The active site itself is embedded in the PP2A domain and covered by the TPR region and the C-terminal inhibitory motif. In its center two catalytic manganese ions are observed (Figures 3A,B).

A superposition of *At*PAPP5 with *Hs*PP5 shows, that *At*PAPP5 mimics the overall structure of the human phosphatase with an r.m.s.d. of 0.70 Å for 365 C_α_-atoms (0.99 Å for 390 C_α_ -atoms). These structures represent the closed state of type 5 serine/threonine protein phosphatases, because the TPR region covers major parts of the active site region. Minor deviations are shown in the arrangement of the TPR region and the helix of the linker region (Figures 3C,D). In contrast to mammalian PP5 structures (Yang et al., 2005; Haslbeck et al., 2015) in our *At*PAPP5 structure the linker region is completely defined by electron density.

**FIGURE 4:**
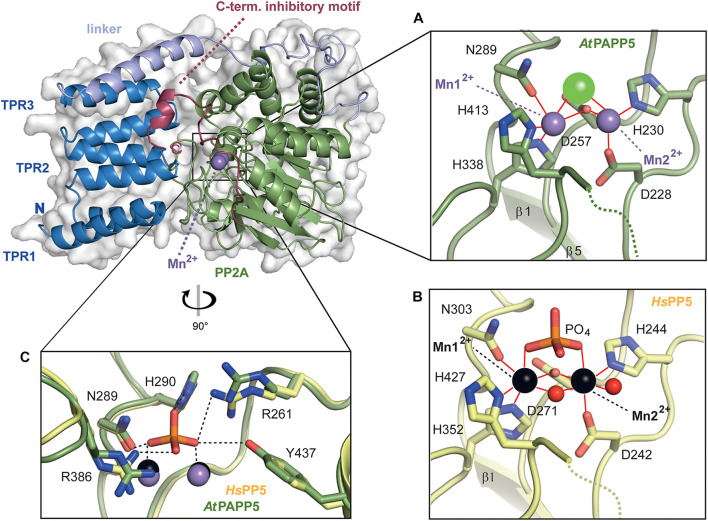
Close-up view of the active site of *At*PAPP5. **(A,B)** Detailed presentation of the coordination spheres of the manganese ions in the phosphatase *At*PAPP5 and *Hs*PP5 (PDB Code: 1S95). Interactions of amino acids with the Mn^2+^ ions (*At*PAPP5: purple, *Hs*PP5: black) are shown in red. A green sphere represents a chloride ion and red spheres water molecules. **(C)** Superposition of the phosphate binding site of *At*PAPP5 and *Hs*PP5 (PDB code: 1S95). The interactions between the phosphate and the amino acids are shown as black dashed lines. The numbering of the amino acids refers to *At*PAPP5.

The local environment of the active site is conserved between *Hs*PP5 and *At*PAPP5 as exemplified by residues D228, H230, D257, N289, H338 and H413 (numbering of amino acids corresponds to *At*PAPP5), which are the coordination partners of the manganese ions. Here, Mn^2+^ ion 1 is tetra-valently coordinated by amino acids D257, N289, H338 and H413, whereas Mn^2+^ ion 2 interacts with amino acids D228, H230 and D257 (Figure 4A). For comparison, a crystal structure of the isolated human PP2A domain (PDB Code: 1S95) (Swingle et al., 2004) shows two additional water molecules. One of them acts like the oxygen atom of the opposing D257 as μ-bridge between the two Mn^2+^ ions thus forming slightly distorted octahedral coordination spheres around the catalytic Mn^2+^ ions (Figure 4B). These μ-bridging water ligands are missing in the *At*PAPP5 structure due to low resolution. Instead, we observe a coordinated chloride ion in our *At*PAPP5 structure (Figures 4A,C). This partly occupied chloride (molecule A: 59%, B: 65%) replaces a phosphate anion that was observed before in the active site of the isolated PP2A domain of HsPP5. Its five interacting ion partners are preserved in *At*PAPP5 (R261, N289, H290, R386, and Y437) and require only some swiveling of the sidechains of R261 and R386 for phosphate binding.

### The C-terminal Inhibitory Motif

**FIGURE 5:**
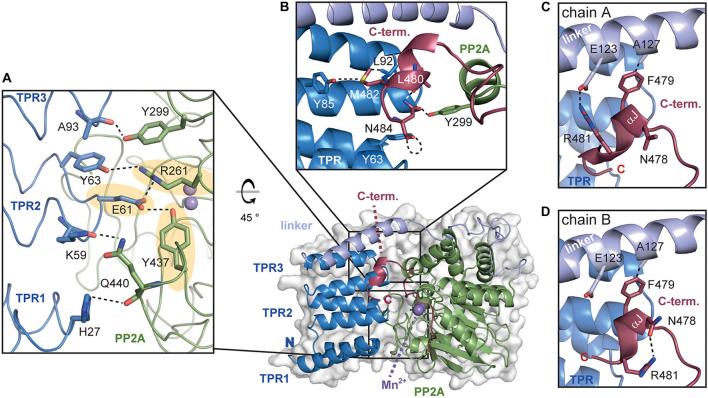
The TPR-PP2A interface of *At*PAPP5. **(A)** The inhibition of the PP2A domain by the TPR region is based on a variety of direct interactions. The hydrogen bonds from E61 to R261 and Y437 (highlighted in yellow), which are also involved in phosphate binding, are depicted in dashed lines. **(B)** The C-terminal helical segment interacts with both the TPR and the PP2A domain. The hydrogen bonding network between C-terminal inhibitory motif and TPR2 as well as the PP2A domain is indicated with dashed lines. **(C,D)** The interactions of the C-terminal helical segment differ between chains A and B. The interactions of the amino acids are represented by dashed black lines.

The *At*PAPP5 structure represents the autoinhibited state, in which both the TPR region as well as the C-terminal inhibitory motif (K471-N484) block the active site and deny access for putative substrate-like phosphopeptides (Figure 5). This mode of self-inhibition was already demonstrated by activity measurements of PP5-type deletion variants (Sinclair et al., 1999; Ryu et al., 2005).

An analysis of the interactions between the TPR and PP2A domain shows that all three TPR repeats form interactions with the PP2A domain (Figure 5A). Here, the prominent hydrogen bonds between the carboxylate group of E61 to Y437 and R261 are of particular interest, since both amino acids are involved in the coordination of the substrate’s phosphate group. Mutation of E61 in *Hs*PP5 to alanine unblocked the active site and raised 10-fold the intrinsic phosphatase activity (Kang et al., 2001). Further stabilization of the inhibited state may be also provided by the interaction between the hydroxyl group of Y63 with R261. These interactions with the phosphate binding site as well as those between the amide oxygen of A93 and the hydroxyl group of Y299 are also conserved in the human and rat PP5 phosphatases. Finally, H27 from TPR1 and K59 from TPR2 form H-bonds with the main chain carbonyl group and the sidechain of Q440 in the PP2A domain, respectively.

The αJ-helix of the C-terminal inhibitory motif is indirectly involved in shielding the active site by stabilizing the closed-state interactions of the PP2A domain with the TPR region (Yang et al., 2005; Haslbeck et al., 2015). In *At*PAPP5, the thioether group of M482 forms interactions with the carbonyl oxygen of Y85 as well as van-der-Waals interactions with L92 (Figure 5B). Unlike mammalian PP5, not only non-polar interactions are found in this region of *At*PAPP5. One prominent interaction is formed by N484, which interacts *via* H-bonds with the carbonyl oxygen of Y63 on TPR1 and Y299 of the PP2A domain. The position of this amino acid is further stabilized by interactions with the amide group of L480. Overall, the polar amino acid N484 is found to bridge both the TPR and the PP2A domains.

The positioning of the αJ-helix is also supported by an interaction with the TPR-PP2A linker. Interestingly, in the two molecules of the asymmetric unit the αJ-helix interacts with different amino acids. Heterogeneity of this helix was also observed in the two PP5 structures from *Rattus norvegicus* (Haslbeck et al., 2015). While the hydrophobic interaction between A127 and the aromatic side chain of F479 is present in both molecules, the polar interactions occur only in molecule A (E123 and R481) and not in molecule B. In the latter, the side chains of R481 and N478 are in H-bonding distance (Figures 5C,D). This interaction pattern might be unique to plant phytochromes since mammalian PP5 lack the involved residues and show no comparable interactions for the C-terminal five amino acids in its structure.

### Conformational Changes of the Tetratricopeptide Repeats Region Activate *At*PAPP5

Heterogeneous interactions of the C-terminal inhibitory motif may indicate that the C-terminus only partly inhibits phosphatase activity and a dynamic equilibrium is formed between active and auto-inhibited *At*PAPP5 forms. This might explain the small, but still measurable activity of the *At*PAPP5 wildtype due to reversible breakage and reformation of interactions between the TPR and the PP2A domains. However, to achieve full phosphatase activity *in vitro* the addition of AA is needed. Size exclusion chromatography (SEC) shows an earlier elution of *At*PAPP5 upon addition of AA, which indicates a larger hydrodynamic radius than the closed, non-activated state of *At*PAPP5 (Figure 1C). To address this issue, we performed CD measurements in the presence of different AA concentrations (Figures 1A,B). These data reveal that the relative α-helical fraction decreases in the presence of AA thus implying partial unfolding of α-helical regions in *At*PAPP5. In order to localize corresponding regions, we applied HDX-MS measurements to AtPAPP5 in the non-actived, closed state as well as in the AA-activated state. Generally, less folded regions are usually characterized by enhanced hydrogen deuterium exchange rates (Figure 1D).

**FIGURE 6:**
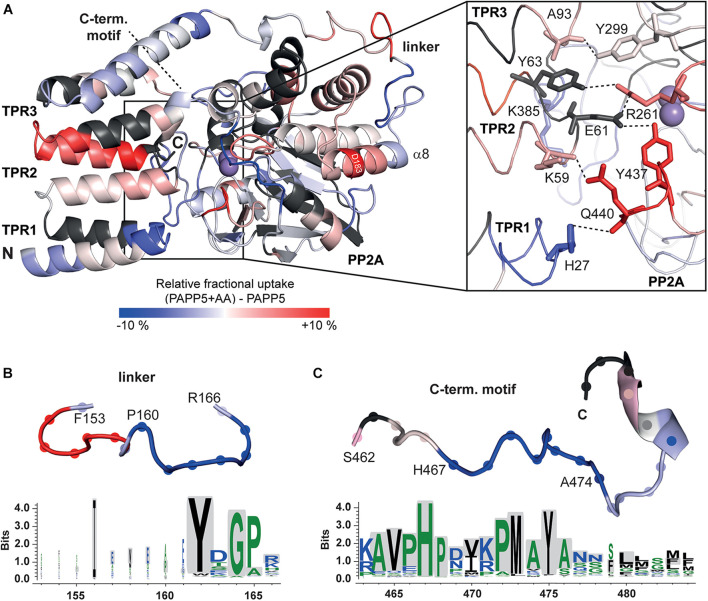
HDX-MS analysis of *At*PAPP5 in blue-white-red representation. **(A)** The difference in deuterium uptake between *At*PAPP5 alone and in the presence of arachidonic acid after 15 s. Regions in red show an increased uptake, while blue structural features represent a reduced exchange rate due to interaction with AA. **(A)** The magnification shows the residues involved in the interaction between TPR and PP2A domains colored according to their uptake. **(B,C)** The linker and the C-terminal motif show both a decreased deuterium uptake compared to the rest of the structure. The conservation of this regions within the PP5 family was generated using the server Weblogo 3.0 (Schneider and Stephens, 1990; Crooks et al., 2004) (using 60 ortholog sequences with a pairwise sequence identity of < 90%). The height of the letters represents the degree of conservation and gray shading marks the sequence of *At*PAPP5.

*A priori* one may predict higher exchange rates for the TPR repeats in the presence of AA due to a disruption of the TPR-PP2A domain interface for opening up the active site of the PP2A domain. Interestingly, the HDX-MS data show only for TPR2 and the beginning of the TPR3 repeat increased exchange rates (Figure 6A). In the AA-activated state H27 of TPR1 experiences surprisingly a reduced H/D exchange rate, whereas its interaction partner within the closed state of *At*PAPP5, Q440, shows increased H/D exchange. Y437 located on the same loop as Q440 shows an increased exchange rate as well. Furthermore, R261 experiences increased exchange so that it can be assumed that its interactions with E61 and Y63 are no longer formed in the AA-actived state. K385 shows a slightly reduced and K59 a slightly increased exchange. Interestingly, some of the interactions between the TPR3 repeat and the PP2A domain apparently remain intact, since Y299 and A93 show no significant changes. It is striking, that only D183 located on the α8-helix has an increased exchange rate. Accordingly, the interactions that D183 had formed to S163 of the linker and K188 of the PP2A domain in the closed state of *At*PAPP5 seem to be broken by addition of AA (Figure 6A).

Two further regions (P160-A165 and H467-A474) stand out due to their significantly reduced uptake rates in the AA-activated state (Figures 6B,C). The first one (P160-A165) is part of the linker between the TPR and PP2A domains. The second region with a reduced exchange rate is part of the C-terminal inhibitory motif (H467-A474). The αJ-helix of the C-terminal motif shows no significant changes in the presence of AA. The last three amino acids of the C-terminus that form both interactions with the TPR and the PP2A domain as well as the corresponding interaction partners are not defined by our HDX-MS analysis (Figure 6C). A comparison of the amino acid sequences of the PP5 phosphatases showed that the linker region contains only three conserved amino acids (Y162, G164, and P165). In contrast, the beginning of the C-terminal inhibitory motif is strictly conserved (Figure 6C).

In order to check for transferability of our *At*PAPP5activation mechanism to other members of the PP5 family, sequence identities of the PP5 phosphatases from different organisms were aligned and analyzed for conservation by the Consurf server (Glaser et al., 2003; Landau et al., 2005). The active site and the interaction interface of the PP2A domain with the TPR region are strongly conserved, compared to the rest of the structure (Supplementary Figure 3). Interestingly, two of the three amino acids of the TPR region that interact with the C-terminal inhibitory motif (Y85 and L92) are conserved in all PP5 phosphatases, as well as E61, which binds in the active site. A complete conservation of the helices that unfold upon addition of AA might not be necessary, as only specific bonds are broken. Furthermore, both regions, which showed a decrease in HDX rate in the presence of AA, are conserved within the PP5 family indicating the general applicability of our activation mechanism. However, the nuclear localization signal is also localized in C-terminal inhibitory motif and could explain the high degree of conservation.

Although the HDX-MS analysis failed to identify unambiguously the AA binding of AtPAPP5, we can summarize (1) that AA binding induces a loss of structure only in the TPR2 repeat of the TPR region and (2) that the C-terminal inhibitory motif stays associated with the TPR region in the active state of *At*PAPP5. One may tentatively suggest that H27 at the tip of TPR1 may represent an interaction partner of AA due to ionic interaction with the AA’s carboxylate group, as several hydrophobic, surface exposed side chains are located near H27 (F24, Y29, F55).

### MD Analysis of a phyB-Phosphopeptide Bound Complex of *At*PAPP5

As the rather local effects of AA-binding to PAPP5 are sufficient for activation of *At*PAPP5, we wondered to what extent the TPR region has to unblock the active site for allowing substrate binding and catalysis. For that purpose, we performed a series of molecular-dynamics simulations, where we sequentially increased the size of the substrate (analog) bound to the di-manganese site in the active center. In the first step, we added a capped phosphoserine residue to the active site, equilibrated this system as NpT ensemble for 80 ns before extending the bound phosphoserine to the phyB D82-S86 peptide phosphorylated at S84. The orientation of the phyB was taken from the complex of human PP5 with a Cdc37 peptide (Oberoi et al., 2016). After further equilibration for 100 ns the *At*PAPP5•peptide complex was elongated to the phyB S80-T89 peptide.

**FIGURE 7:**
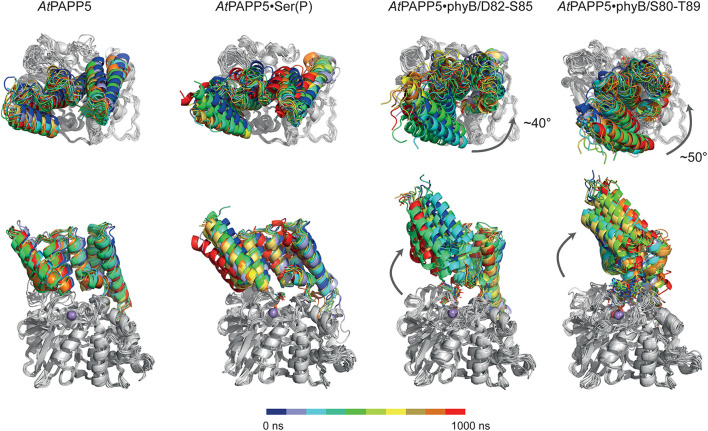
MD simulation of *At*PAPP5 and its complexes to phosphorylated phyB peptides. Ten snapshots of a representative 1 μs trajectory are shown with the PP2A domains superposed on each other to highlight displacements of the TPR region. The upper row shows a view mostly along the linker helix between the TPR region and PP2A domain; here a counter-clockwise movement of the TPR region relative to the PP2A domain is apparent upon binding of phyB peptides. The lower row depicts a perpendicular view and shows partial off-swiveling of the TPR region to open up the peptide-binding channel of *At*PAPP5.

**FIGURE 8:**
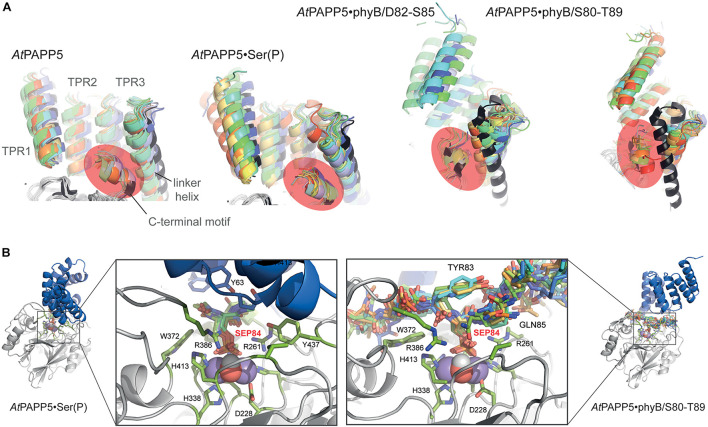
Effect of phosphopeptide binding to the TPR-PP2A domain arrangement in *At*PAPP5. **(A)** The TPR regions of 10 MD snapshots (colored) are shown in comparison to the crystal structure of *At*PAPP5 (black). The C-terminal inhibitory motif is highlighted in orange. In the phyB peptide complexes the motif moves closer to the N-terminal portion of the linker helix. **(B)** Active site shown for 10 MD snapshots of the *At*PAPP5•N-acetyl-SEP-NMe (left) and the *At*PAPP5•phyB/S80-T89 complexes.

MD trajectories for the closed state of AtPAPP5 show only minor differences for the association of the TPR region and the PP2A domain when comparing to the crystal structure of *At*PAPP5 and other PP5-type phosphatases (Figure 7). Only the interactions of Q440 with H27 and K59, which were observed in the crystal structure, are apparently weaker in the MD ensembles (Supplementary Table 4) due to intrusion of bridging water molecules. Docking of the short phosphoserine peptide N-acetyl-SEP-NMe into the active site of AtPAPP5 causes apparently no major structural adaptions of the closed state. The center-of-mass (COM) distances between the TPR region and the PP2A domain increases only slightly from ∼28 Å to 29–30 Å (Supplementary Figure 4). Nevertheless, the H-bonding interactions Y437/E61, Q440/K59 and Y299/Y63 of the substrate-free state are broken in the TPR-PP2A interface. Elongating the phosphopeptide to the S84-phosphorylated phyB peptide D82-S85 severely distorts the TPR-PP2A interface by triggering a rotation of the TPR-region by ∼40° relative to the PP2A domain and the linking helix (Figure 7) and an increase of the COM-COM distance of both domains to 32–34 Å. As a consequence, main chain interactions between G64-S65 from TPR2 and K385-R386, which are a feature of the closed states, are disrupted. Despite the large-scale conformational changes the C-terminal inhibitory motif remains associated during simulation time with the TPR region (Figure 8A). Given the mostly hydrophobic interactions of this motif with TPR3 residues, it is notable that its only salt bridge in the closed state between the C-terminal carboxyl group of N484 and K59, which is apparently only transiently formed during MD simulations of the closed state (Supplementary Table 4) and not defined at all for molecule B of the *At*PAPP5 crystals, forms a new salt bridge to the side chain of K120 from the linker helix. Finally, in the complex with the capped phyB decapeptide S80-T89 there is a slight increase of the TPR-region rotation to ∼50° without a change of the COM-COM distance. The MD trajectories of this AtPAPP5 complex tend to form again more homogeneous ensembles in the now open state than the AtPAPP5•phyB/D82-S85 complexes, because the former exert a lesser degree of conformational variation for the TPR-PP2A domain arrangement (Figure 7 and Supplementary Figure 4).

The phosphoserine S84 of the phyB peptides is coordinated to the dimanganese center and forms ionic interactions with the sidechain of R261; the protonated H290 forms a hydrogen bond to the OG atom of the phosphoserine and apparently acts as general acid during catalysis (Figure 8B). Despite being sandwiched between the TPR and PP2A domains, the conformation of the bound phosphopeptides follows the *in-out-in-out* rule for peptide binding by PP5-type phosphatases as previously suggested (Oberoi et al., 2016), where the phosphoserine adopts the inward orientation toward the active site.

## Discussion

Protein phosphorylation and dephosphorylation are commonly used as on-/off-switches in the regulation of cellular activities in almost all organisms. The transfer of a phosphate group to a protein often exerts profound effects on its structure and functional properties (Krebs, 1994). Accordingly, strict control of kinases and phosphatases often depends on regulatory domains or subunits, which undergo structural changes due to substrate binding (Uhrig et al., 2013). Studies with phosphatases of the PP5 family revealed that activation can be achieved by addition of AA or proteolytic digestion of their TPR region and 13 C-terminal amino acids (Kang et al., 2001). Our interaction studies with the *At*PhyB variants mimicking the phosphorylated state of the NTE could show that the PSM alone is sufficient for the interaction and dephosphorylation by *At*PAPP5 in the presence of AA. Additional interactions between *At*PAPP5 and HKR of the C-terminal module may strengthen interactions between *At*PhyB and *At*PAPP5 but are not essential for the interaction with the phosphorylated state of *At*PhyB.

Activity control by fatty acids is not restricted to phosphatases of the PP5 family. One example is the Rho kinase from *Homo sapiens*, which is blocked by a C-terminal autoinhibitory subunit, where addition of AA activates the kinase (Araki et al., 2001). Likewise, an inhibitory effect is known for the smooth muscle myosin light chain phosphatase (SM-PP1M). A dissociation of the catalytic subunit is triggered by AA, resulting in a reduced dephosphorylation efficiency (Gong et al., 1992; Somlyo and Somlyo, 1994). In plants, AA and other polyunsaturated fatty acids inhibit protein phosphatase MP2C (Baudouin et al., 1999), whereas in animals AA stimulate PP2Cα (Klumpp et al., 1998). Like for 2C-type phosphatases the binding mode and activation mechanism by AA are poorly understand for PP5 phosphatases. Previous data point to a disruption of the interaction between TPR and the active site of the PP2A domain (Skinner et al., 1997). Mutations of the amino acid E61 from TPR2, which interacts with the catalytic site and L480, which stabilizes the C-terminal helix, increase the phosphatase activity. Interestingly, the mutation of the amino acid N477 of the C-terminal inhibitory motif, which is not directly involved in interactions between the PP2A and TPR regions, shows a 50% decreased sensitivity to activation by AA (Kang et al., 2001). This contradicts the hypothesis that AA merely binds to the TPR region and directly effects a destabilization of the α-helical structure of the TPR region by interactions with its hydrophobic amino acids.

AA-driven structural transformations were initially indicated by soaking experiments of *At*PAPP5 crystals, which caused rapid dissolution of the crystals. Our HDX-MS experiments show that after AA-addition accessibility changes occur indeed in the TPR region as well as in the linker and the C-terminal inhibitory motif. The end of the TPR2 repeat and the beginning of the TPR3 repeat possess increased exchange rates, indicating that interactions between the second and third repeat get broken and at least partial unfolding of this region takes place. Accordingly, the amino acids of the PP2A domain, which interact with the TPR1 and TPR2 repeats, show increased exchange rates, indicating a dissociation of these TPR-repeats from the active site surface of the PP2A domain as indicated by SEC due to the increased hydrodynamic radius of the AtPAPP5⋅AA complex. In contrast, the TPR3 repeat apparently still interacts with the PP2A domain as its H/D exchange rate is almost unaltered. Furthermore, the beginning of the C-terminal inhibitory motif and the end of the linker connecting the TPR and PP2A domains exert strongly reduced exchange rates upon addition of AA. Since these areas are the only ones in *At*PAPP5 with decreased conformational dynamics in the *At*PAPP5⋅AA complex, they may directly interact with each other. This notion is corroborated by our extensive MD simulations with docked phyB phosphopeptides. Here, we also observe dissocation of TPR1 from the PP2A domain and an increased exposure of TPR2 to solvent (Figure 7), whereas the C-terminal inhibitory motif still packs to TPR3 and the linker helix despite a swivel motion of 40–50° for the TPR region. Apparently, small-molecule activators of mammalian PP5 can act allosterically in an analogous manner as AA (Haslbeck et al., 2015). These synthetic compounds have been found to bind to the PP2A domain close to TPR1 and cause a slight swiveling motion of the TPR region. Although our HD/X MS-data could not unambiguously reveal the AA binding site of *At*PAPP5, we found a significant decrease of accessibility for H27 of TPR1 upon AA addition. We suggest a scenario for PP5-type phosphatases, where allosteric activators like AA first weaken the TPR-PP2A interactions at the peripheral site of the closed state. The loss of TPR-PP2A interactions allows then a directed movement of the TPR domain relative to the PP2A domain. The resulting open state still provides parts of the TPR region, especially TPR3, for interaction with substrate peptides. In this scenario, the specificity for substrate phosphopeptides, such as the N-terminal, phosphorylated regions around S84 and S86 of phyB, would not only been conferred by the PP2A domain (Oberoi et al., 2016), but partly also by TPR3. Finally, during inactivation of the PP5-type phosphatase the C-terminal inhibitory motif may act then as a return spring for the back-positioning of the TPR region to the closed state conformation.

In order to prove the transferability of our activation mechanism for *At*PAPP5 to other members of the PP5 family, sequence identities of the PP5 phosphatases from different organisms were aligned and analyzed for conservation by the Consurf server (Glaser et al., 2003; Landau et al., 2005). The active site and the interaction interface of the PP2A domain with the TPR region are strongly conserved, compared to the rest of the structure (Supplementary Figure 3). Interestingly, two of the three amino acids of the TPR region that interact with the C-terminal inhibitory motif (Y85 and L92) are conserved in all PP5 phosphatases, as well as E61, which binds in the active site. A complete conservation of the helices that unfold upon addition of AA might not be necessary, as only specific bonds are broken. Furthermore, both regions, which showed a decrease in HDX rate in the presence of AA, are conserved within the PP5 family indicating the general applicability of our activation mechanism. However, the nuclear localization signal is also localized in C-terminal inhibitory motif and could explain the high degree of conservation.

In plants the role of AA is still controversial. It has so far only been found in the plastids of algae and mosses, where it is produced by chain lengthening of the linoleic acid and subsequent desaturation. Interestingly, the exogenous treatment of plants with AA, a lipid that is usually derived from animals, triggers programmed cell death as well as plant defense mechanisms against insect pathologs (Bostock et al., 1981; Knight et al., 2001; García-Pineda et al., 2004).

## Data Availability Statement

The datasets presented in this study can be found in online repositories. The names of the repository/repositories and accession number(s) can be found below: http://www.wwpdb.org/, 7OBE.

## Author Contributions

SH performed the experiments. SH and L-OE analyzed the data, wrote the manuscript, and designed the research. Both authors reviewed the manuscript.

## Conflict of Interest

The authors declare that the research was conducted in the absence of any commercial or financial relationships that could be construed as a potential conflict of interest.

## Publisher’s Note

All claims expressed in this article are solely those of the authors and do not necessarily represent those of their affiliated organizations, or those of the publisher, the editors and the reviewers. Any product that may be evaluated in this article, or claim that may be made by its manufacturer, is not guaranteed or endorsed by the publisher.

## Footnotes

1
https://github.com/Amber-MD/pytraj
