# Supplementary material for: Conformational Change of Tetratricopeptide Repeats Region Triggers Activation of Phytochrome-Associated Protein Phosphatase 5
Source: Front Plant Sci. 2021 Oct 14;12:733069. doi: 10.3389/fpls.2021.733069 (PMC8551457; doi:10.3389/fpls.2021.733069)
Supplement: Supplementary file 1 [file Data_Sheet_1.PDF]

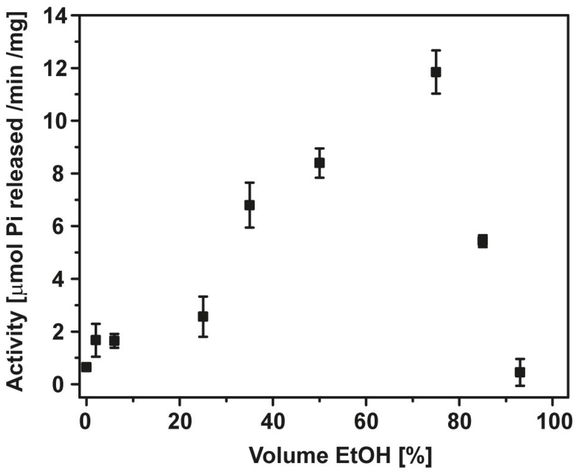

Supplementary Figure S1. Influence of ethanol on the phosphatase activity of *At*PAPP5. The activity (mean ± SD; n=3) was measured in the presence of 50 mM p-nitrophenyl phosphate (pNPP) and varying ethanol concentrations.

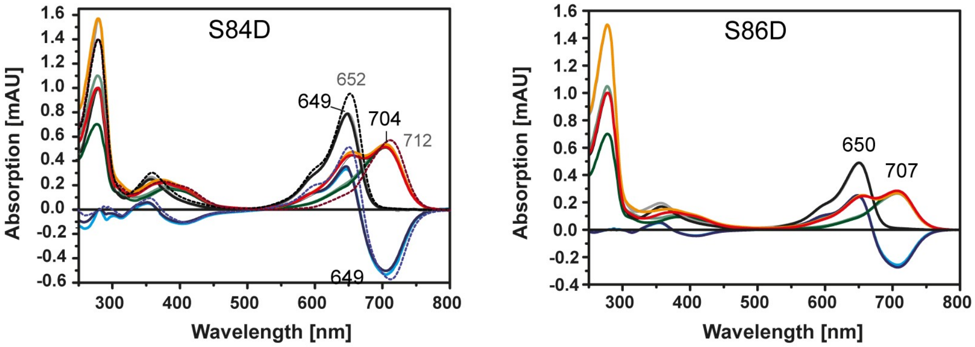

Supplementary Figure S2. UV/Vis spectra of the variants *At*PhyB/S84D and *At*PhyB/S86D. The spectrum of the P_r, the P_{fr} and the calculated P_{fr} states are shown in black, red and green, respectively; the difference spectrum is depicted in blue. The corresponding spectra of the *At*PhyB variants in the presence of *At*PAPP5 and AA are depicted in grey, orange, light green and turquoise. In the S84D panel, the wild type spectra of the P_r, the P_{fr} and the calculated P_{fr} states are shown for comparison (dotted lines in black, dark red and blue).

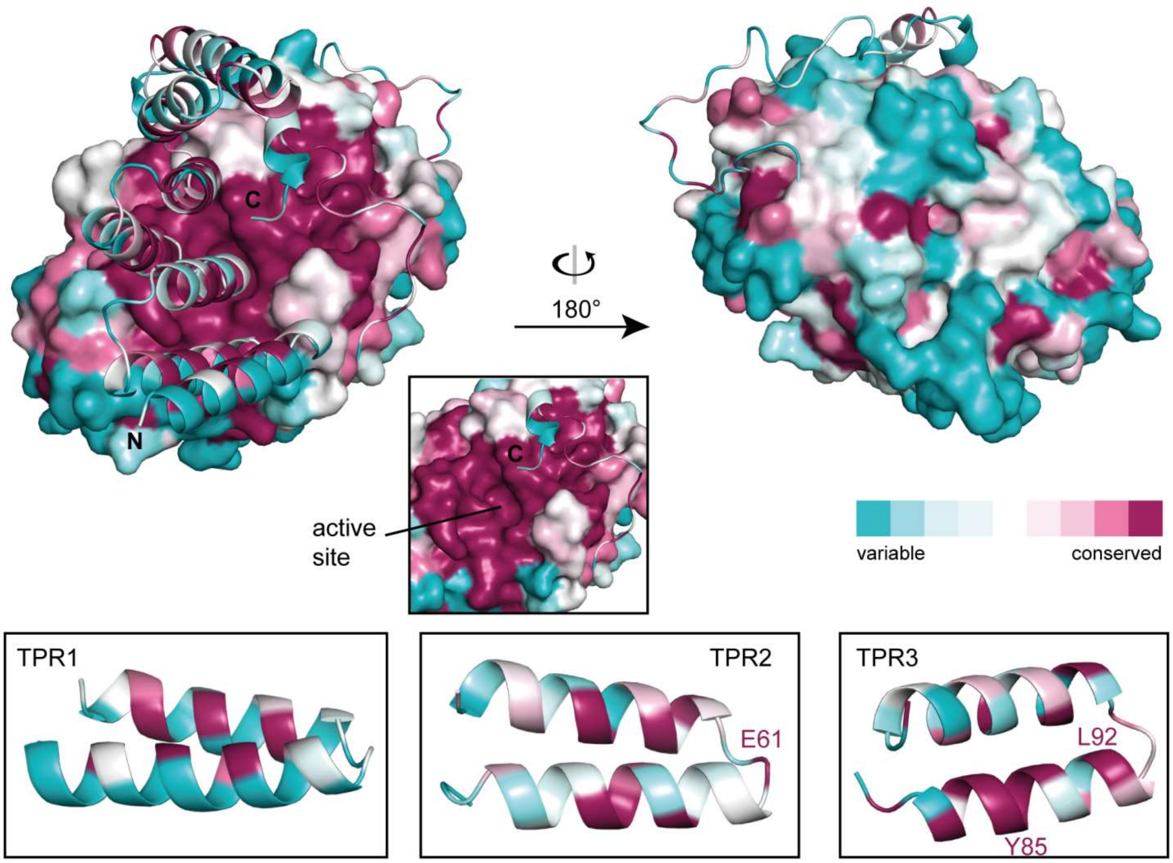

Supplementary Figure S3. Coloration of the PP5 phosphatases according to their degree of conservation. The TPR domain, the linker and the C-terminal inhibiting motif are shown with their secondary structures and the PP2A domain as a surface model. The magnification shows the manganese binding site, which is strictly conserved in all PP5 phosphatases. The colour scale from turquoise over white to magenta indicates the degree of conservation, whereby magenta marks conserved amino acids. E61 binds into the active site of the PP2A domain and is strictly conserved in all PP5 phosphatases. Furthermore, the residues Y85 and L92, which form interactions with the C-terminal motif, are also conserved. The degree of conservation was calculated by Consurf (Glaser et al., 2003; Landau et al., 2005) by using 60 orthologous sequences with a sequence identity <90%.

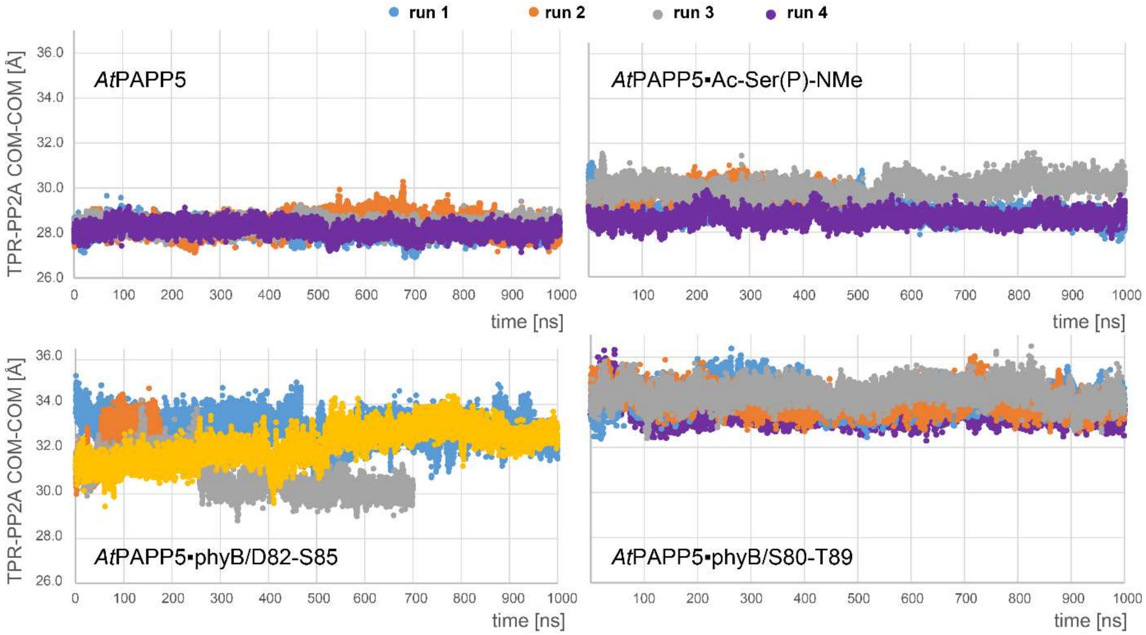

Supplementary Figure S4. MD trajectories of AtPAPP5 alone (upper left) or in complex with phosphorylated phyB peptides. Centre-Of-Mass (COM) distances between the TPR region (5-142) and PP2A domain (205-N484)
