# Supplementary material for: Conformational Change of Tetratricopeptide Repeats Region Triggers Activation of Phytochrome-Associated Protein Phosphatase 5
Source: Front Plant Sci. 2021 Oct 14;12:733069. doi: 10.3389/fpls.2021.733069 (PMC8551457; doi:10.3389/fpls.2021.733069)
Supplement: Supplementary file 2 [file Table_1.DOCX]

Supplementary Table S1**: Oligonucleotides used for DNA amplification.**

| **protein/primer name** | **sequence** | **restriction enzymes** |
| --- | --- | --- |
| ***At*PAPP5(136-472)** |  |  |
| *At*PAPP5(136-472)_fwd | 5’-CGGATTACATATGCCGGATGCAACCCGTA A | *Nde*I |
| *At*PAPP5(136-472)_rv | 5’-GCAAAATTGGATCC**TTA**CATCGGTTTAACA TCAGGATGCG | *Bam*HI |
| ***At*PhyA(973-1122)** |  |  |
| *At*PhyA(873-1122)_fwd | 5’-CGTAGGGGGATCCGCAACTTGCCAGCCAT GAGCTGC | *Bam*HI |
| *At*PhyA(873-1122)_rv | 5’-GCTTCGTGCGGCCGC**TTA**CTTGTTTGCTG CAGCGAGTTCCG | *Not*I |
| ***At*PhyB(1-651)** |  |  |
| *At*PhyB(1-651)_fwd | 5’-GGTCGCACATGTCGTATGGTTTCCGGAGT CGG | *Pci*l |
| *At*PhyB(1-651)_rv | 5’-GCACGTCTGCAGTTAATGGTGATGGTGAT GATG | *Pst*I |
| ***At*PhyB_S84D** |  |  |
| *At*PhyB(1-651)_S84D_fwd | 5’-GAAATCATTCGACTAC*GAT*CAATCACTC |  |
| *At*PhyB(1-651)_S84D_rv | 5’-CCTGATTCGCCGGATTGTTCG |  |
| ***At*PhyB_S86D** |  |  |
| *At*PhyB(1-651)_S86D_fwd | 5’-GACTACTCACA*GAT*CTCAAAACG |  |
| *At*PhyB(1-651)_S86D_rv | 5’-GAATGATTTCCCTGATTCG |  |
