# Supplementary material for: Conformational Change of Tetratricopeptide Repeats Region Triggers Activation of Phytochrome-Associated Protein Phosphatase 5
Source: Front Plant Sci. 2021 Oct 14;12:733069. doi: 10.3389/fpls.2021.733069 (PMC8551457; doi:10.3389/fpls.2021.733069)
Supplement: Supplementary file 3 [file Table_2.DOCX]

Supplementary Table S2**: Molecular dynamics analyses of *At*PAPP5 complexes.**

| **MD System** | **run** | **equi [ns]** | **prod [ns]** | **box x** | **box y** | **box z** | **water** | **Na^+^** | **Cl^-^** |
| --- | --- | --- | --- | --- | --- | --- | --- | --- | --- |
| AtPAPP5 | 1 | 100 | 1000 | 100.56 | 100.75 | 100.52 | 31718 | 58 | 57 |
| AtPAPP5 | 2 | 100 | 1000 |  |  |  |  |  |  |
| AtPAPP5 | 3 | 100 | 1000 |  |  |  |  |  |  |
| AtPAPP5 | 4 | 100 | 1000 |  |  |  |  |  |  |
| AtPAPP5•NAc-Ser(p)-NMe | 1 | 100 | 1000 | 100.58 | 100.76 | 100.53 | 31720 | 58 | 54 |
| AtPAPP5•NAc-Ser(p)-NMe | 2 | 100 | 500 |  |  |  |  |  |  |
| AtPAPP5•NAc-Ser(p)-NMe | 3 | 100 | 1000 |  |  |  |  |  |  |
| AtPAPP5•NAc-Ser(p)-NMe | 4 | 100 | 1000 |  |  |  |  |  |  |
| AtPAPP5•NAc-Asp-Tyr-Ser(p)-Gln-Ser-NMe | 1 | 100 | 1000 | 100.52 | 100.71 | 100.47 | 31654 | 58 | 53 |
| AtPAPP5•NAc-Asp-Tyr-Ser(p)-Gln-Ser-NMe | 2 | 100 | 180 |  |  |  |  |  |  |
| AtPAPP5•NAc-Asp-Tyr-Ser(p)-Gln-Ser-NMe | 3 | 100 | 700 |  |  |  |  |  |  |
| AtPAPP5•NAc-Asp-Tyr-Ser(p)-Gln-Ser-NMe | 4 | 100 | 1000 |  |  |  |  |  |  |
| AtPAPP5•NAc-Ser-Phe-Asp-Tyr-Ser(p)-Gln-Ser-Leu-Lys-Thr-NMe | 1 | 100 | 1000 | 110.37 | 110.34 | 110.61 | 42625 | 58 | 54 |
| AtPAPP5•NAc-Ser-Phe-Asp-Tyr-Ser(p)-Gln-Ser-Leu-Lys-Thr-NMe | 2 | 100 | 1000 |  |  |  |  |  |  |
| AtPAPP5•NAc-Ser-Phe-Asp-Tyr-Ser(p)-Gln-Ser-Leu-Lys-Thr-NMe | 3 | 100 | 1000 |  |  |  |  |  |  |
| AtPAPP5•NAc-Ser-Phe-Asp-Tyr-Ser(p)-Gln-Ser-Leu-Lys-Thr-NMe | 4 | 100 | 1000 |  |  |  |  |  |  |
