# Supplementary material for: Conformational Change of Tetratricopeptide Repeats Region Triggers Activation of Phytochrome-Associated Protein Phosphatase 5
Source: Front Plant Sci. 2021 Oct 14;12:733069. doi: 10.3389/fpls.2021.733069 (PMC8551457; doi:10.3389/fpls.2021.733069)
Supplement: Supplementary file 4 [file Table_3.DOCX]

Supplementary Table S3**: Average distances within AtPAPP5 and its complexes as obtained by MD simulations.**

| **Measured distances [Å]** | **Exp. Xtal dist.** | **Mean apo** | **Std apo** | **Mean Ser(P)** | **Std Ser(P)** | **Mean D82-S85** | **Std D82-S85** | **Mean S80-S89** | **Std S80-S89** |
| --- | --- | --- | --- | --- | --- | --- | --- | --- | --- |
| **PP2A-TPR** |  |  |  |  |  |  |  |  |  |
| R261CZ:E61CD | 3.58 | 4.66 | 0.26 | 5.14 | 1.25 | 10.93 | 0.72 | 14.39 | 1.29 |
| R261NH:Y63OH | 3.79 | 9.46 | 1.31 | 11.27 | 2.55 | 13.25 | 0.91 | 13.34 | 0.60 |
| Y299OH:Y63OH | 3.48 | 3.90 | 0.60 | 6.45 | 0.96 | 10.08 | 0.79 | 12.50 | 1.98 |
| Y299OH:A93O | 2.30 | 3.07 | 0.31 | 3.42 | 0.99 | 6.67 | 0.90 | 8.26 | 1.53 |
| K385NZ:E62CD | 4.80 | 4.80 | 0.69 | 5.62 | 0.59 | 12.76 | 1.40 | 25.58 | 11.01 |
| K385O:S65N | 2.95 | 3.05 | 0.02 | 3.39 | 0.59 | 9.52 | 1.31 | 19.80 | 10.48 |
| R386O:G64N | 3.75 | 3.12 | 0.06 | 3.27 | 0.42 | 11.02 | 1.04 | 18.26 | 10.13 |
| Y437OH:E61OE | 3.81 | 2.88 | 0.12 | 5.54 | 1.75 | 10.90 | 0.29 | 12.78 | 2.46 |
| Q440OE1:K59NZ | 3.14 | 4.66 | 0.61 | 6.82 | 3.49 | 12.41 | 0.12 | 16.45 | 1.62 |
| Q440O:H27ND1 | 2.90 | 4.12 | 0.19 | 7.16 | 4.00 | 16.48 | 0.17 | 22.27 | 2.45 |
| **C-tail-TPR** |  |  |  |  |  |  |  |  |  |
| N477N:I298O | 2.92 | 3.58 | 1.00 | 3.37 | 0.60 | 3.59 | 0.97 | 3.21 | 0.34 |
| N478ND2:E123OE | 9.67 | 4.20 | 1.26 | 6.29 | 1.39 | 9.55 | 2.06 | 9.30 | 2.21 |
| F479arom:A127CB | 4.39 | 5.19 | 0.28 | 5.31 | 0.93 | 7.10 | 0.96 | 7.68 | 2.52 |
| R481NH:N478OD1 | 2.73 | 4.01 | 0.49 | 5.47 | 0.78 | 6.99 | 2.39 | 6.63 | 1.44 |
| F483arom:Y85OH | 4.15 | 5.23 | 0.43 | 5.97 | 1.78 | 5.33 | 0.96 | 6.60 | 2.64 |
| F483arom:F104arom | 5.89 | 5.61 | 0.47 | 7.01 | 2.70 | 6.46 | 0.77 | 8.30 | 3.75 |
| F483arom:F479arom | 9.31 | 6.49 | 0.48 | 8.37 | 2.79 | 6.09 | 0.21 | 6.91 | 1.98 |
| N484Oend:K59NZ | 2.93 | 4.81 | 0.84 | 8.67 | 1.84 | 8.25 | 0.23 | 9.21 | 1.52 |
| ASN484Oend:R119NE | 14.48 | 13.37 | 1.12 | 8.85 | 3.31 | 7.99 | 0.35 | 7.03 | 1.60 |
| ASN484Oend:K120NZ | 9.75 | 8.37 | 1.26 | 5.04 | 1.98 | 4.14 | 0.26 | 3.97 | 0.63 |
| **Active site** |  |  |  |  |  |  |  |  |  |
| H290ND1:D260OD2 | 2.84 | 2.72 | 0.01 | 2.72 | 0.00 | 2.73 | 0.02 | 2.73 | 0.00 |
| **Peptide-PAPP5** |  |  |  |  |  |  |  |  |  |
| ASP82OD:R386NH2 | n.a. |  |  |  |  | 10.05 | 0.23 | 9.73 | 3.91 |
| ASP82OD:R386NH1 | n.a. |  |  |  |  | 10.44 | 0.58 | 9.38 | 4.13 |
| TYR83OH:M94O | n.a. |  |  |  |  | 8.38 | 1.45 | 4.01 | 0.75 |
| SEP84O3P:GLN85N | n.a. |  |  |  |  | 2.96 | 0.05 | 3.00 | 0.06 |
| SEP84OG:R261NH2 | n.a. |  |  | 4.19 | 2.22 | 2.97 | 0.27 | 3.79 | 0.94 |
| SEP84OG:H290NE2 | n.a. |  |  | 2.96 | 0.11 | 3.02 | 0.02 | 2.94 | 0.07 |
| SEP84N:Y299OH | n.a. |  |  |  |  | 5.27 | 0.63 | 4.89 | 0.54 |
| GLN85O:R261NH | n.a. |  |  |  |  | 3.94 | 0.33 | 3.78 | 0.25 |
| GLN85O:Y437OH | n.a. |  |  |  |  | 3.16 | 0.69 | 3.62 | 0.79 |
| SER86O:R481NH | n.a. |  |  |  |  | 10.72 | 2.78 | 11.03 | 5.64 |
